# Adenoid Cystic Carcinoma of the External Auditory Canal

**DOI:** 10.1016/S1808-8694(15)31394-X

**Published:** 2015-10-17

**Authors:** Carolina Pimenta Carvalho, Alano Nunes Barcellos, Daniel Caldeira Teixeira, Juliano de Oliveira Sales, Rui da Silva Neto

**Affiliations:** 1 MD, second-year student at specialization program on ENT; 2 MD, third-year student at specialization program on ENT; 3 MD, first-year student at specialization program on ENT; 4 MD, specialist on ENT and head and neck surgery, Preceptor at the ENT Specialization Program at Hospital Socor; 5 MD, specialist on ENT, Head of the ENT Service at Hospital Israel Pinheiro, IPSEMG, Preceptor at the ENT Specialization Program at Santa Casa de Belo Horizonte. Hospital Socor

**Keywords:** carcinoma, adenoid cystic, salivary glands

## Abstract

Adenoid cystic carcinoma is a rare tumor originating from the salivary glands, especially when arise the external auditory canal. This tumor has high rate of perineural invasion and metastasis, then must be treated with aggressive surgery combined with postoperative radiation. We report a case of an adenoid cystic carcinoma arising the external auditory canal of 77 years old female patient, who complained hypoacusis and pain. She was treated by radical mastoidectomy and radiotherapy

## INTRODUCTION

Adenoid cystic carcinoma is a rare disease, as it accounts for 3–10% of the salivary gland tumors and 1–4% of all head and neck neoplasms.^1^, ^2^, ^3^, ^4^ It is found more often in the smaller salivary glands^1^, ^2^, ^3^, but may also appear in lacrimal glands, tracheobronchial tree, breasts, esophagus, and external ear canal (EEC).^2^

Tumors in the EEC are quite rare, as glands are the preferential site for adenoid cystic carcinomas. Symptoms are pain, hypoacusis, and slow-growth nodule. It is characterized by perineural invasion and distal metastasis, as in other adenoid cystic tumors.^5^, ^6^

## CLINICAL CASE

**Figure 1:**
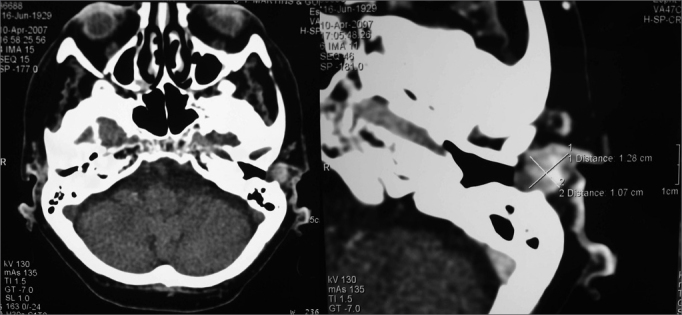
Adenoid Cystic Carcinoma in Left External Ear Canal - Axial view CT scans of temporal bones (with and without contrast enhancement)

A 77-year-old female patient came to our service complaining of intense left otalgia present for the past 6 months, accompanied by mild hypoacusis and a slow-growth lump in the left outer ear meatus. During physical examination a tumor of fibroelastic consistence was found in the upper wall of the external ear canal, occupying nearly the entire diameter of the meatus. The patient felt pain during palpation. CT scans of the temporal bones were ordered (Fig. 1) and a solid, round, average density lump enhanced by contrast was found. The borders were partially defined, and the neoplasm measured 1.46 cm in its widest point. The tumor was located in the superior-anterior portion of the left EEC. Intracanal resection surgery was performed and pathology tests classified the tumor as a cribriform adenoid cystic carcinoma with compromised margins. A modified radical mastoidectomy was then performed and the entire ear meatus was removed, including the region of the tragus. This time the pathology tests showed no presence of disease. The patient was referred to additional treatment with radiotherapy four weeks after surgery.

## DISCUSSION

EEC tumors are rare and only 20% have glandular origins. Most are squamous cell carcinomas. However, among the glandular tumors, adenoid cystic carcinomas are the most prevalent. The characteristics are the same as encountered in small salivary gland ACC,^5^, ^6^ with silent growth, local recurrence, perineural invasion, and late distal metastasis.^1^, ^3^, ^4^, ^5^

There is controversy in the literature as to gender prevalence. Triantafillidou K et al. found it to be more frequently found in women, while Lucia A et al. have seen increased incidence among males.^1^, ^5^ The tumor may appear at any age, but incidence peaks in the 5th and 6th decades of life.^1^ Symptoms are pain, hypoacusis, otorrhea and EEC nodule, and the tumor usually grows for years before the patient is diagnosed.^5^, ^6^

Diagnosis is done through pathology examination. Fine needle aspiration has been used to assist in the preoperative diagnosis.^2^

Radical mastoidectomy coupled with ear canal resection and specimen freezing to assess margins and perineural invasion is the treatment of choice.^2^, ^5^, ^6^ Post-operative radiotherapy is also recommended to help control local tumor recurrence (86% against 11% when only surgery is done)^1^, ^3^, ^4^. Some authors recommend radiotherapy only in advanced tumors when there is skull base invasion, neck metastasis, perineural invasion, solid histological type, and relapsing tumors.^4^, ^5^, ^6^

Neck clearance is offered only if positive nodules are found in the neck, which happens approximately in 4% of the cases.^1^, ^3^, ^4^ Distal metastasis are more frequent and occur in 48% of the patients, mainly when the primary tumor was not completely removed.^4^ Lungs, kidneys, and vertebrae are the most commonly involved sites.^2^ In spite of the metastatic sites, patients tend to live for a long time with the disease, thus increasing the importance of controlling the tumor locally to reduce morbidity and maintain reasonable levels of quality of life for the patient.^3^

Prognosis is worse when the tumor relapses locally (32% of the cases), when there is perineural, parotid or bone involvement, and in cases when margins are positive and the tumor is of the solid histological type.^1^, ^2^, ^4^, ^6^

## CONCLUSION

Adenoid cystic carcinoma is an uncommon tumor that rarely involves the external ear canal. It must be removed using radical procedures to increase the chances of controlling the disease locally and even reduce the occurrence of distal metastasis.
